# Long-Term Outcomes Following Surgical Repair for Post-cholecystectomy Biliary Strictures

**DOI:** 10.7759/cureus.64405

**Published:** 2024-07-12

**Authors:** Venkkatesh Sreepathi, Karthikeyan Srinivasan, Sastha Ahanatha Pillai, Villalan Ramasamy, M. B. Krishna Prasad Chowdary, Thamarai Kannan Murugesan, Padmanabhan Subbareddiar

**Affiliations:** 1 Department of Surgical Gastroenterology, Madurai Medical College, Madurai, IND

**Keywords:** hepp-couinaud, hepaticojejunostomy, benign biliary stricture, bile duct injury, cholecystectomy

## Abstract

Introduction: Bile duct injuries (BDIs) are a serious complication of cholecystectomy. Strictures that form after major injuries ultimately require surgical repair. This study aimed to analyse our experience with the surgical repair of post-cholecystectomy biliary strictures (PCBS).

Methods: Patients who underwent surgical repair for PCBS between January 2013 and March 2020 were retrospectively reviewed. The strictures were classified using the Bismuth system. Delayed repair with Roux-en-Y hepaticojejunostomy was performed using the Hepp-Couinaud technique. Outcomes were graded according to McDonald’s criteria. Statistical analysis was performed to identify factors influencing the outcomes.

Results: Sixty-eight patients underwent repair for PCBS. Forty-five patients presented within one month and eight patients presented late after six months. Presenting symptoms were jaundice, external biliary fistula, biliomas, cholangitis and peritonitis. Portal hypertension was present in two patients. The median interval for definitive repair was 22 weeks. The median hospital stay was 9.5 days. Eighteen patients had postoperative complications. One patient had postoperative mortality due to uncorrectable coagulopathy. With a median follow-up of 54 months, successful outcomes were achieved in 61 (90%) patients. Four patients had anastomotic strictures evident at two, four, five and eight years after repair. Portal hypertension and postoperative complications were the variables associated with poor outcomes.

Conclusion: BDIs following cholecystectomy are a devastating complication. Surgical repair for biliary strictures yields durable long-term outcomes with early identification and timely referral to a tertiary care centre where standardized techniques for biliary reconstruction are followed.

## Introduction

Bile duct injuries (BDIs) occurring after cholecystectomy are an iatrogenic catastrophe that requires a multidisciplinary approach for optimum management. The incidence of BDIs following open cholecystectomy is 0.1% to 0.2% and is 0 to 1% for laparoscopic cholecystectomy as reported in various studies [1,2].

A major BDI leads to the formation of a benign biliary stricture (BBS). Surgical repair for BBSs has been shown to have better long-term outcomes compared to endoscopic and radiological management [3]. Non-surgical management is an option in patients with intact biliary ductal continuity following partial strictures and in those with long-standing BBSs with secondary biliary cirrhosis (SBC) as a bridge to liver transplant.

The blood supply to the extrahepatic bile duct depends predominantly on two arterial plexuses. One is the 9 and 3 o'clock arteries (axial vessels) between the right hepatic artery and posterior pancreaticoduodenal and gastroduodenal arteries. The other one is at the hilar plate on the inferior surface of the liver [4,5]. Because of the rich blood supply at the hilum, biliary reconstruction performed at the hilum provides the best chance at good long-term outcomes. This is most commonly performed by incorporating the left duct using the Hepp-Couinaud approach [6,7].

In this study, we aimed to analyse the outcomes after surgical repair of post-cholecystectomy biliary strictures (PCBS) in patients referred to our institution and to identify the factors affecting the outcome.

## Materials and methods

A prospectively maintained database of 135 patients with post-cholecystectomy bile duct injuries who were managed by the Department of Surgical Gastroenterology, Government Rajaji Hospital, Madurai, Tamil Nadu, from January 2013 to March 2020 was reviewed retrospectively. Patients with minor leaks who were managed conservatively and did not develop biliary strictures (n=29) were excluded. Patients who were lost to follow-up (n=22) and those who underwent immediate intraoperative repair (n=16) were also excluded. The remaining patients (n=68) who underwent surgical repair for biliary strictures were included for analysis in this study.

Data regarding patient demographics, indication and technique of cholecystectomy, time of recognition of injury, presentation, interventions before definitive repair, type of stricture, the interval between index surgery and repair, diagnostics, surgical management and outcome were recorded.

Patients who presented with uncontrolled bile leaks or bilomas underwent drainage, percutaneous or surgical as needed. Those who had controlled biliary fistulas were managed conservatively for six to eight weeks. Endoscopic retrograde cholangiopancreatography (ERCP) with stenting of the bile duct was performed when the fistula persisted for more than eight weeks. Patients who presented with cholangitis underwent biliary drainage either by ERCP or percutaneous transhepatic biliary drainage (PTBD). All the patients had a minimum waiting period of 12 weeks from the injury before planning a definitive repair. They were subjected to magnetic resonance cholangiopancreatography (MRCP) to identify the type of stricture and classified according to Bismuth classification [8,9]. An upper GI endoscopy was done to look for the presence of features of portal hypertension. In selected patients, Doppler ultrasound and CT of the abdomen were performed to look for portal vein thrombosis and atrophy-hypertrophy complex. The definitive procedure included Roux-en-Y hepaticojejunostomy (RYHJ) using single-layer interrupted 3-0 vicryl sutures with the extension of the stoma to the left duct as described by Hepp [6] and was aimed at achieving an effective anastomosis of >2 cm. For high strictures with more than one duct at the hilum, either the ducts were approximated and treated as a single duct for anastomosis or separate anastomoses were made [10]. An intra-abdominal drain was always placed in the Morrison's pouch and no trans-anastomotic stents were used. Patients were followed up with liver function test (LFTs) four weeks post-repair and then every six months for two years. Radiological imaging, either ultrasound or MRCP was performed when LFTs were deranged. Postoperative complications were defined as short-term if occurring within 30 days and long-term if occurring after 30 days of surgery. Follow-up was done as outpatients with check-up visits and telephone interviews. The length of follow-up was calculated from the time of definitive repair at our centre. Outcomes were classified according to the McDonald grading system [11] based on symptoms, biochemical tests and radiological imaging at the last follow-up. Those undergoing re-intervention after definitive repair were given grade D, and their further follow-up is not included in the analysis. Grades A and B were considered as successful and grades C and D were taken as unsatisfactory outcomes.

Statistical analysis

An analysis was performed to investigate the relationship of risk factors like the type of stricture, the interval between injury and definitive repair, presence of cholangitis episodes preoperatively, presence of features of portal hypertension, and postoperative complications with outcome grades. The collected data were analysed with IBM SPSS Statistics for Windows, version 29.0 (IBM Corp., Armonk, NY). To describe the descriptive statistics frequency analysis, percentage analysis was used for categorical variables and medians and ranges were used for continuous variables. To find the predictors of the poor outcome, binary logistic regression analysis was used. To find the significance in qualitative categorical data, the chi-square test was used. If the expected cell frequency was less than 5 in 2×2 tables, Fisher's exact test was used. In all these statistical tools, a probability value of <0.05 was considered significant (see the Appendices for a detailed analysis).

## Results

Patient characteristics and presentations

**Figure 1:**
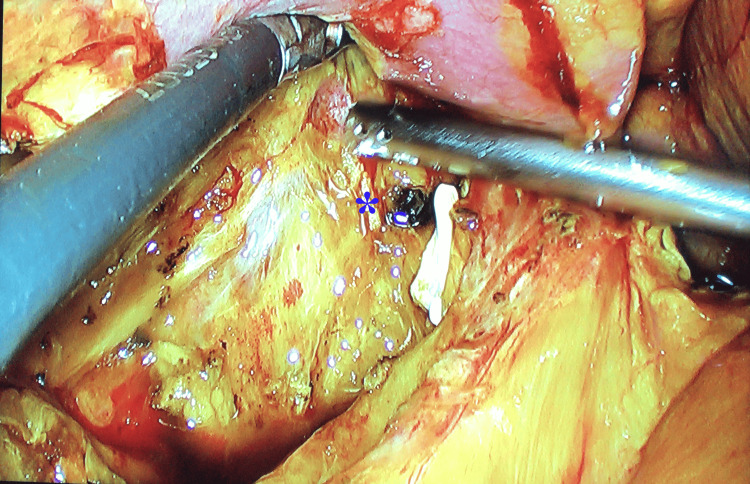
Clip noted at the distal common bile duct with transected proximal end (asterisk) during laparoscopic lavage for biliary peritonitis

Between January 2013 and March 2020, 68 patients underwent surgical repair for post-cholecystectomy benign biliary strictures. There were 48 (71%) females and 20 (29%) males and the age ranged from 17 to 66 years (median, 36 years). Cholecystitis was present in 44 (65%) patients and 24 (35%) had uncomplicated cholelithiasis. Index surgery was laparoscopic cholecystectomy in 49 (72%) patients, laparoscopic converted to open cholecystectomy in 19 (28%) patients. Among 68 patients, 45 (66%) patients presented within one month. The rest of them presented after one month; among them, 15 (22%) patients presented within six months and 8 (12%) patients presented late after six months (Table 1). Jaundice was the presenting symptom in 31 (46%) patients and external biliary fistula (EBF) in 21 (31%). Two patients with EBF had undergone ERCP and stenting elsewhere and the rest of them were managed conservatively. All the EBFs healed within eight weeks. Biliomas were present in 12 (18%) patients and were managed with ultrasound-guided percutaneous drainage. One patient with bilioma underwent additional laparoscopic lavage and drainage. Episodic cholangitis was the presenting symptom in 10 (15%) patients, of whom six underwent ERCP stenting and the rest four underwent PTBD. Seven (10%) patients presented with peritonitis requiring surgical management; four underwent laparoscopic lavage (Figure 1) and three underwent laparotomy and drainage. A total of 32 (47%) patients had interventions prior to definitive repair.

**Table 1:** Characteristics of patients ^a^Six had jaundice and three had fistula along with a bilioma. ^b^Four had jaundice along with cholangitis.

	n (%)
Age, median (range), years	36 (17-66)
Female	48
Male	20
Laparoscopic, n (%)	49 (72)
Laparoscopic cholecystectomy converted to open, n (%)	19 (28)
Time of presentation, n (%)	
<1 month	45 (66)
1-6 months	15 (22)
>6 months	8 (12)
Presenting symptom, n (%)	
Jaundice	31 (46)
External biliary fistula	21 (31)
Bilioma	12^a^ (18)
Cholangitis	10^b^ (15)
Peritonitis	7 (10)

**Figure 2:**
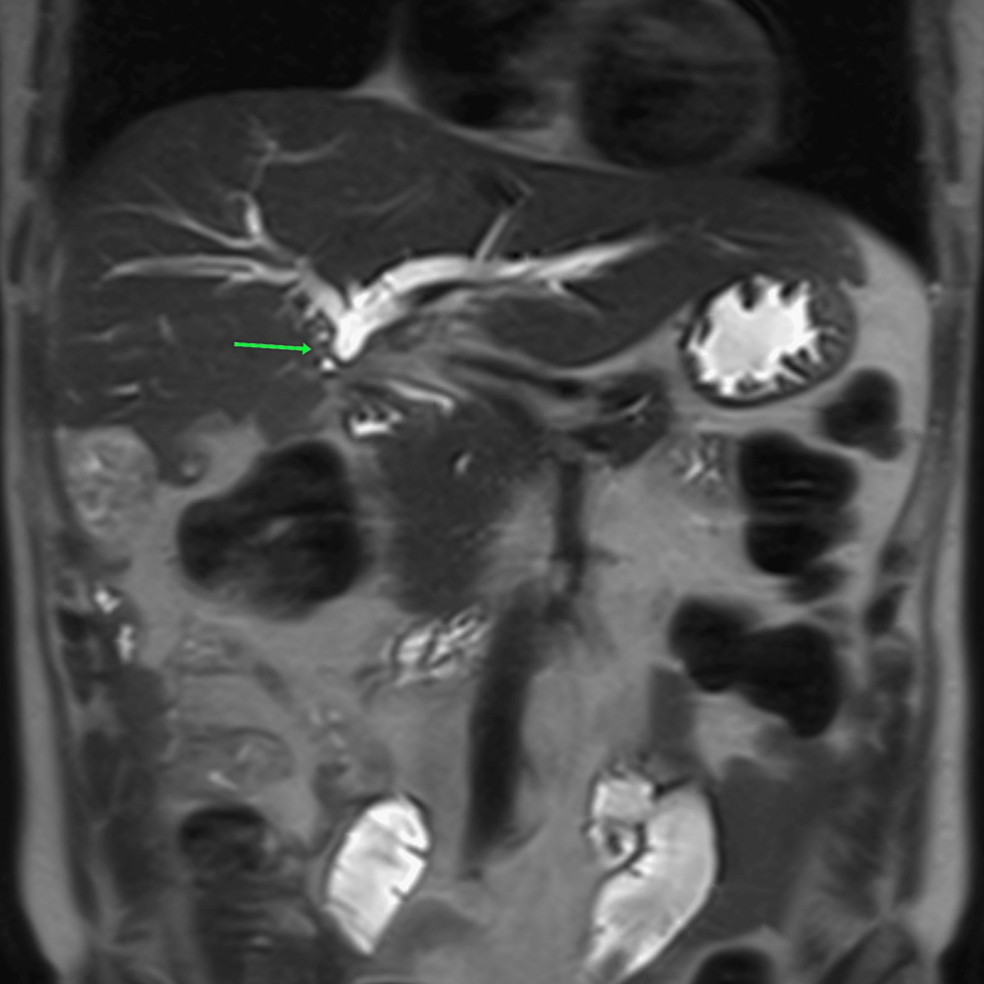
Bismuth type 3 stricture on magnetic resonance cholangiopancreatography

**Figure 3:**
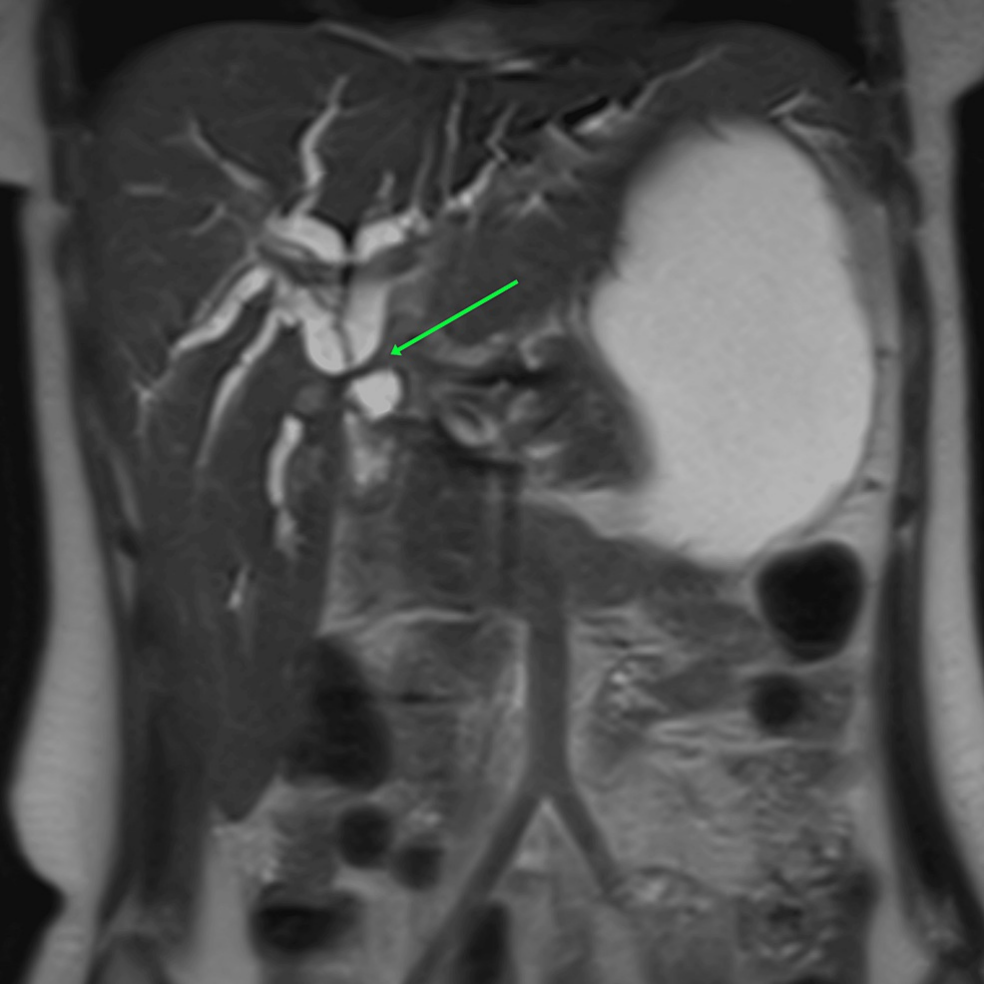
Bismuth type 4 stricture on magnetic resonance cholangiopancreatography

Surgical repair was electively planned after a minimum interval of 12 weeks. The median interval between index cholecystectomy and definitive repair was 22 weeks. Strictures were graded using MRCP according to the Bismuth classification (Table 2). Types of injuries were as follows: type 1 in 4 (6%), type 2 in 28 (41%), type 3 in 33 (49%) and type 4 in 3 (4%) patients (Figures 2-3). Upper GI endoscopy revealed esophageal varices in two patients. Both the patients had splenomegaly and one of them had symptomatic hypersplenism.

**Table 2:** Interventions, type of stricture, and intervals ^a^One had undergone both percutaneous and surgical intervention.

	n (%)
Interventions before definitive surgery, n (%)	
Percutaneous	16^a^ (24)
Endoscopic	9 (13)
Surgical	8 (12)
None	36 (53)
Type of stricture, n (%)	
Type 1	4 (6)
Type 2	28 (41)
Type 3	33 (49)
Type 4	3 (4)
Interval between injury to definitive surgery, n (%)	
12-24 weeks	42 (62)
25-48 weeks	21 (31)
>48 weeks	5 (7)

Surgery and complications

**Figure 4:**
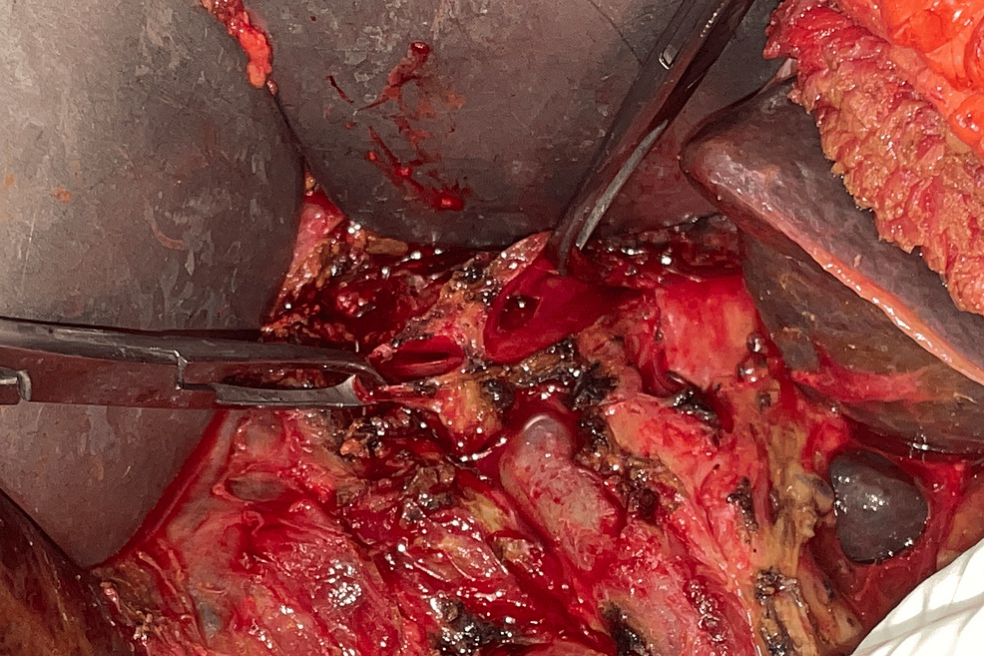
Type 4 stricture with the roof of the confluence destroyed

**Figure 5:**
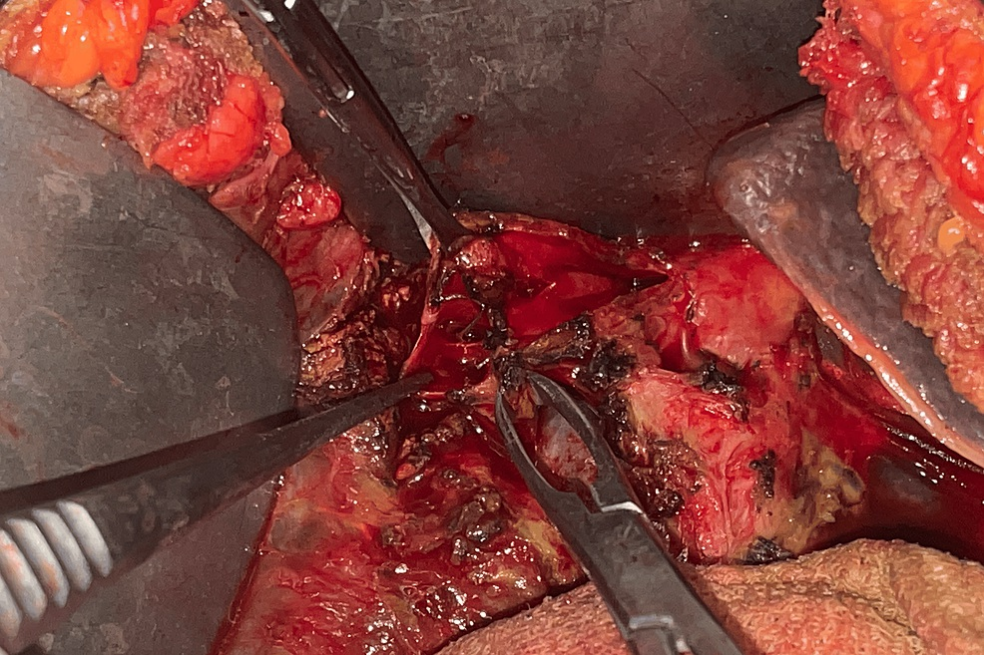
Septoplasty performed between the left and right duct to use it as a single stoma

**Figure 6:**
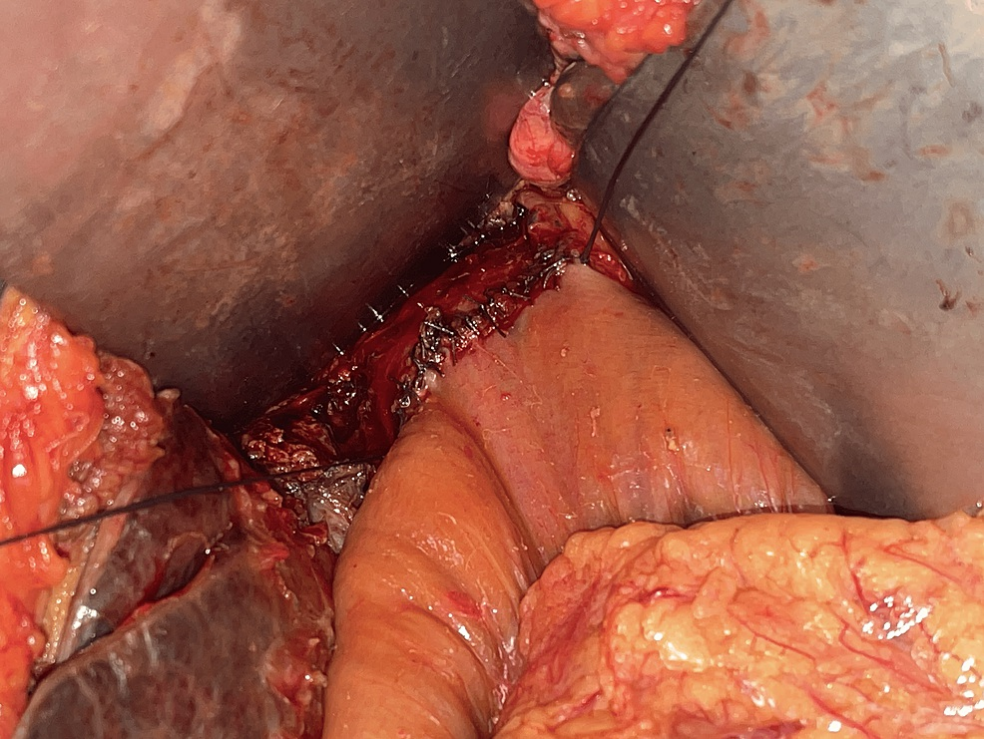
Hepaticojejunostomy in a Bismuth type 4 stricture

In all the patients, the repair was carried out with a side-to-side RYHJ. Anastomosis was extended over to the left duct in 66 patients. In two patients with type 4 stricture, septoplasty was performed between the left and right duct to make it a single duct for anastomosis (Figures 4-6). In one patient with type 4 stricture, the two ducts were not amenable for approximation; hence, two separate anastomoses were made. Splenectomy with devascularisation was added along with bilio-enteric anastomosis in one patient having features of portal hypertension. An effective stoma size of >2 cm was achieved in 67 (98.5%) patients. The median hospital stay was 9.5 days (range: 7-18 days).

Postoperative complications were seen in 18 (26%) patients. The most common complication was wound infection, seen in 10 (15%) patients. Six (9%) patients had bile leaks that settled with conservative management. Among the two patients with features of portal hypertension preoperatively, one developed ascites postoperatively that was managed with diuretics. The other one developed uncorrectable coagulopathy and had a fatal outcome (mortality of 1.47%) (Table 3).

**Table 3:** Complications ^a^One with anastomotic stricture had a liver abscess and another had portal hypertension.

Short-term complications	n (%)
Wound infection	10 (15)
Anastomotic leak	6 (9)
Ascites	1 (2)
Death	1 (2)
Long-term complications	n (%)
Anastomotic stricture^a^	4 (6)
Incisional hernia	2 (3)
Recurrent cholangitis	1 (2)
Portal hypertension	1 (2)
Secondary biliary cirrhosis	1 (2)
Liver abscess	1 (2)

Follow-up and outcome analysis

Sixty-seven patients were followed up on an outpatient basis with a median follow-up period of 54 months (range: 18-116 months). Thirty-one patients (46%) had a follow-up of two to five years, 30 (44%) patients had more than five years of follow-up and six (9%) patients had less than two years of follow-up (Table 4).

**Table 4:** Follow-up after definitive repair ^*^One patient died in the postoperative period.

Duration of follow-up after definitive repair	n (%)^*^
<24 months	6 (9)
24–60 months	31 (46)
>60 months	30 (44)

Eight patients (12%) developed long-term complications, of whom four (6%) had anastomotic strictures. One patient developed SBC, one had recurrent episodes of cholangitis and two had incisional hernias. The time of anastomotic stricture in the four patients was two, four, five and eight years after definitive repair. Successful outcomes, McDonald's grades A and B, were achieved in 58 (86%) and 3 (4%) patients, respectively. Unsatisfactory outcomes, grades C and D, were seen in 2 (3%) and 5 (7%) patients, respectively (Table 5).

**Table 5:** Grades and outcomes LFT, liver function test

Grade	Description	n (%)
A: Excellent	No symptoms. Normal LFT	58 (86)
B: Good	No symptoms. Mild elevation in the LFT	3 (4)
C: Fair	Cholangitis and pain. Elevation in the LFT	2 (3)
D: Poor	Requiring endoscopic or surgical management and postoperative death	5 (7)

On analysis of the relationship between various factors and outcomes, the presence of portal hypertension at the time of surgery and the development of postoperative complications had a statistically significant influence on the outcome (p=0.009 and p=0.012, respectively). Type of stricture, interventions prior to definitive management, preoperative cholangitis episodes and the interval between initial surgery and definitive repair had no influence on the outcome.

## Discussion

A BDI following cholecystectomy remains a dreaded complication. The successful management of bile duct injuries depends upon multiple factors such as early identification, evaluation, timing of intervention, procedure chosen, experience of the centre and general condition of the patient. Surgical repair of biliary strictures can be complicated by bile leak, anastomotic strictures and biliary cirrhosis. The main purpose of the repair is to achieve adequate biliary drainage without causing postoperative symptoms or liver function disorders.

This article describes the outcomes in a consecutive series of 68 patients undergoing surgical repair for post-cholecystectomy biliary stricture at our institution over seven years. Index cholecystectomy in all the cases in our study was initiated with laparoscopy, of which a few had open conversion. Cholecystitis was present in two-thirds of the patients in our study where local factors like inflammation and haemorrhage could have led to the injury. The rest of them had an uncomplicated cholelithiasis indicating anatomical orientation as a major problem. The misinterpretation of either normal or variant anatomy plays a pivotal role in the causation of bile duct injury [2,12,13].

Histologically, the bile duct heals by collagen deposition and scar formation. When the scar contracture occurs, it leads to stricture formation [14]. Surgical repair with a wide bilio-enteric anastomosis with mucosa-to-mucosa approximation is essential for getting optimal results. A wide stoma >2 cm helps in maintaining the biliary drainage even after the scar contracture.

Most of the BDIs are missed at the initial surgery, with only 16%-25% being identified intraoperatively [15]. Early recognition of injury is a key step in the management. Those presenting within a month, in our study, had external biliary fistulas, undrained collections (biliomas) or peritonitis whereas those presenting late had jaundice with or without cholangitis. Repair in the early postoperative period is associated with poor outcomes due to the presence of sepsis and inflammation [16,17].

Acute phase management by controlling sepsis and with a minimum wait period of 12 weeks before attempting surgical repair was followed in our centre [18]. Thirty-two (47%) patients had percutaneous, endoscopic or surgical interventions prior to the definitive repair, but it had no statistically significant effect on the outcome in our study (p=0.42).

Timing of repair is considered critical. A delay in repair can be complicated by the development of SBC. SBC has been reported to occur as early as 20 weeks to 6 months in various studies [19]. Along with the prolonged injury-repair interval, the degree of obstruction also influences the development of SBC. Portal hypertension in the absence of cirrhosis can be present and was postulated to be related to the alteration in hepatic hemodynamics in the presence of biliary obstruction [20]. Portal hypertension has been reported in 7% to 20% of patients with post-cholecystectomy benign biliary stricture at the time of referral [21-23]. Agarwal et al. reported good outcomes with hepaticojejunostomy for post-cholecystectomy BBSs complicated with portal hypertension with a patent portal vein and recommended shunt surgery prior to RYHJ when the portal vein is blocked [24]. In our study, the presence of portal hypertension at the time of repair, rather than the injury-repair interval had a significant negative impact on the outcome (p=0.009).

Evaluation of the strictures with MRCP has become the gold standard. Although the sensitivity for detection of BBSs is low (67%), specificity remains high (98%) [25]. Most groups now perform angiography (either CT or MR) to evaluate associated vascular injuries and pseudoaneurysms that helps in the management of acute BDIs. The level of BDIs ascends as a result of infection and ischemia, more so if an associated vascular injury is present [7,26]. We did not use angiography in the preoperative evaluation of BBSs as we followed delayed repair. Bismuth classification that was introduced before the laparoscopic era is still the most commonly used classification for BBSs. This classification is useful in anticipating the difficulty of the repair and predicts the long-term outcomes. High strictures (types 3 and 4) and low strictures (types 1 and 2) were seen in 53% and 47% patients, respectively, in our study with the majority being type 3. Several studies [21,22] have emphasized the level of stricture as a risk factor for failure. We could not, however, find any statistically significant relationship between the level of stricture and outcomes (p=0.637).

Surgical repair with RYHJ is considered safe with many series reporting <1% perioperative mortality [27]. In patients with coexisting portal hypertension, mortality has been reported to be as high as 23%-80% [21,23]. The morbidity is usually in the form of anastomotic leak, wound infection and cholangitis. The incidence of postoperative wound infections is higher if biliary drains and stents are present at the time of repair. Our study reported a complication rate of 26% that is comparable to the 15%-43% reported from various specialized centres [28], and the development of postoperative complications led to poor long-term outcomes (p=0.012).

Satisfactory outcomes following RYHJ are reported to be 80%-90% when the repair is undertaken at tertiary care centres [11,29,30]. Anastomotic strictures can occur as late as 10 years, but the majority develop within two years. Hence, a longer follow-up duration is recommended [17,31]. Most of the patients in our study had a follow-up of more than two years and three out of four anastomotic strictures were seen after two years. Therefore, a long-term follow-up of at least 10 years following surgical repair cannot be overemphasized.

This study has several limitations. The analysis was performed retrospectively with the sample size being small for multivariate analysis. The evaluation of concomitant vascular injury and its effect on the outcome was not analysed. Although the surgical repair was performed on an elective basis, we did not investigate the effect of preoperative bilirubin, haemoglobin and albumin on postoperative outcomes. The stage of liver fibrosis at the time of surgery was not taken into consideration. Some follow-ups were telephonic consultations that may be inadequate. Finally, the long-term quality of life was not analysed.

## Conclusions

Post-cholecystectomy bile duct injuries have devastating consequences and care must be taken to prevent them. The key to the successful management of these injuries lies in early identification and timely referral to tertiary hepato-biliary centres, where the required investigative modalities and surgical expertise are available. Our results indicate that surgical repair offers excellent long-term outcomes for post-cholecystectomy BBSs. Surgical management with an anastomosis at the hilum (Hepp-Couinaud approach), a wider stoma (>2 cm) and a delayed repair with a long-term follow-up is imperative to manage these strictures.

## Appendices

Statistical analysis

The collected data were analysed using IBM SPSS Statistics for Windows, version 29.0 (IBM Corp., Armonk, NY). To describe the descriptive statistics frequency analysis, percentage analysis was used for categorical variables and the means and SDs were used for continuous variables. To find the significant difference between the bivariate samples in independent groups, the independent sample t-test was used. To find the predictors of the poor outcome, binary logistic regression analysis was used with enter method. To find the significance in qualitative categorical data, a chi-square test was used; similarly, if the expected cell frequency was less than 5 in 2×2 tables, then the Fisher's exact was used. In all these statistical tools, a probability value of .05 was considered significant.

**Table 6:** Comparison of genders in relation to the outcome using Fisher’s exact test ^#^No statistical significance at p > 0.05 level. ꭓ^2^=2.890, p=0.182 (>0.05), which shows that there was no statistically significant effect of gender on the outcome.

			Outcome		Total	ꭓ^2^-value	p-value
			Good	Poor			
Gender	Female	Count	45	3	48	2.890	0.182^#^
		%	73.8%	42.9%	70.6%		
	Male	Count	16	4	20		
		%	26.2%	57.1%	29.4%		
Total		Count	61	7	68		
		%	100.0%	100.0%	100.0%		

**Table 7:** Comparison of interventions prior to definitive management in relation to the outcome using Fisher’s exact test ^#^No statistical significance at p > 0.05 level. ꭓ^2^=1.244, p=0.429 (>0.05), which shows that there was no statistically significant effect of interventions prior to definitive management on the outcome.

			Outcome		Total	ꭓ^2^-value	p-value
			Good	Poor			
Interventions prior to definitive management	Absent	Count	30	5	35	1.244	0.429^#^
		%	49.2%	71.4%	51.5%		
	Present	Count	31	2	33		
		%	50.8%	28.6%	48.5%		
Total		Count	61	7	68		
		%	100.0%	100.0%	100.0%		

**Table 8:** Comparison of the presence of portal hypertension at the time of definitive repair in relation to the outcome using Fisher’s exact test **Highly statistically significant at p < 0.01 level. ꭓ^2^=17.957, p=0.009 (<0.01), which shows that the presence of portal hypertension at the time of definitive repair had a highly statistically significant effect on the outcome.

			Outcome		Total	ꭓ^2^-value	p-value
			Good	Poor			
Presence of portal HTN at the time of definitive repair	No	Count	61	5	66	17.957	0.009**
		%	100.0%	71.4%	97.1%		
	Yes	Count	0	2	2		
		%	0.0%	28.6%	2.9%		
Total		Count	61	7	68		
		%	100.0%	100.0%	100.0%		

**Table 9:** Comparison of the types of stricture in relation to the outcome using Pearson’s chi-square test ^#^No statistical significance at p > 0.05 level. ꭓ^2^=1.701, p=0.637 (>0.05), which shows that the type of stricture had no statistically significant effect on the outcome.

			Outcome		Total	ꭓ^2^-value	p-value
			Good	Poor			
Type of stricture	1	Count	3	1	4	1.701	0.637^#^
		%	4.9%	14.3%	5.9%		
	2	Count	26	2	28		
		%	42.6%	28.6%	41.2%		
	3	Count	29	4	33		
		%	47.5%	57.1%	48.5%		
	4	Count	3	0	3		
		%	4.9%	0.0%	4.4%		
Total		Count	61	7	68		
		%	100.0%	100.0%	100.0%		

**Table 10:** Comparison of intervals (I & S) in relation to the outcome using Pearson’s chi-square test ^#^No statistical significance at p > 0.05 level. ꭓ^2^=2.023, p=0.073 (>0.05), which shows that there was no statistically significant effect of interval (I & S) on the outcome. "Interval (I & S)" is the interval between injury and surgical repair.

			Outcome		Total	ꭓ^2^-value	p-value
			Good	Poor			
Interval (I & S)	12-24 weeks	Count	39	3	42	5.243	0.073^#^
		%	63.9%	42.9%	61.8%		
	25-48 weeks	Count	19	2	21		
		%	31.1%	28.6%	30.9%		
	>48 weeks	Count	3	2	5		
		%	4.9%	28.6%	7.4%		
Total		Count	61	7	68		
		%	100.0%	100.0%	100.0%		

**Table 11:** Comparison of preoperative cholangitis episodes in relation to the outcome using Fisher’s exact test ^#^No statistical significance at p > 0.05 level. ꭓ^2^=2.023, p=0.330 (>0.05), which shows that there was no statistically significant effect of preoperative cholangitis episodes on the outcome.

			Outcome		Total	ꭓ^2^-value	p-value
			Good	Poor			
Preoperative cholangitis episodes	Absent	Count	47	7	54	2.023	0.330^#^
		%	77.0%	100.0%	79.4%		
	Present	Count	14	0	14		
		%	23.0%	0.0%	20.6%		
Total		Count	61	7	68		
		%	100.0%	100.0%	100.0%		

**Table 12:** Comparison of postoperative complications in relation to the outcome using Fisher’s exact test *Statistical significance at p < 0.05 level. ꭓ^2^=8.103, p=0.012 (<0.05), which shows that postoperative complications had a statistically significant effect on the outcome.

			Outcome		Total	ꭓ^2^-value	p-value
			Good	Poor			
Postoperative complications	Absent	Count	48	2	50	8.103	0.012*
		%	78.7%	28.6%	73.5%		
	Present	Count	13	5	18		
		%	21.3%	71.4%	26.5%		
Total		Count	61	7	68		
		%	100.0%	100.0%	100.0%		

**Table 13:** Results for age in relation to the outcome using the independent sample t-test ^#^No statistical significance at p > 0.05 level. t=1.079, p=0.284 (>0.05), which shows no statistically significant difference at p > 0.05 level.

Variable	Outcome	N	Mean	SD	t-value	p-value
Age	Good	61	36.4	11.3	1.079	0.284^#^
	Poor	7	41.1	6.7		

**Table 14:** Results for interval in relation to the outcome using the independent sample t-test ^#^No statistical significance at p > 0.05 level. t=0.425, p=0.672 (>0.05), which shows no statistically significant difference at p > 0.05 level.

Variable	Outcome	N	Mean	SD	t-value	p-value
Interval	Good	61	56.9	27.0	0.425	0.672^#^
	Poor	6	62.0	38.7		

**Table 15:** Comparison of factors' influence on the outcome using binary logistic regression ^*^Significant at p < 0.05. ^#^No statistical significance at p > 0.05. "Postoperative complications" showed statistically significant difference at p < 0.05 level, whereas all other variables showed no statistically significant difference at p > 0.05 level and Nagelkerke R-square value = 0.532. "Interval (I & S)" is the interval between injury and surgical repair.

Variables in the equation								
	B	SE	Wald's	df	p-value	Exp(B)	95% CI for Exp(B)	
							Lower	Upper
Age	-.021	.062	.115	1	0.734^#^	.979	.868	1.105
Gender	1.789	1.643	1.186	1	0.276^#^	5.983	.239	149.651
Interventions prior to definitive management	-.700	1.335	.275	1	0.600^#^	.497	.036	6.792
Type of stricture	-1.942	1.199	2.622	1	0.105^#^	.143	.014	1.505
Interval (I & S)	.022	.037	.341	1	0.559^#^	1.022	.950	1.099
Preoperative cholangitis episodes	-20.436	8372.146	.000	1	0.998^#^	.000	0.000	
Postoperative complications	4.418	2.019	4.788	1	0.029^*^	82.895	1.585	4335.510
Interval	.010	.021	.217	1	0.641^#^	1.010	.969	1.053
Constant	-.400	3.950	.010	1	0.919^#^	.670		
